# Phase I clinical trial of multiple-peptide vaccination for patients with advanced biliary tract cancer

**DOI:** 10.1186/1479-5876-12-61

**Published:** 2014-03-07

**Authors:** Atsushi Aruga, Nobuhiro Takeshita, Yoshihito Kotera, Ryuji Okuyama, Norimasa Matsushita, Takehiro Ohta, Kazuyoshi Takeda, Masakazu Yamamoto

**Affiliations:** 1 Department of Gastroenterological Surgery, Tokyo Women's Medical University, 8-1 Kawada-cho, Shinjuku-ku, Tokyo 162-8666, Japan; 2 Institute of Advanced Biomedical Engineering and Science, Tokyo Women's Medical University, 8-1 Kawada-cho, Shinjuku-ku, Tokyo 162-8666, Japan; 3 Department of Immunology, Juntendo School of Medicine, 2-1-1 Hongo, Bunkyo-ku, Tokyo 113-8421, Japan

**Keywords:** Cancer vaccine, Peptide vaccine, Immunotherapy, Biliary tract cancer

## Abstract

**Background:**

The prognosis of patients with advanced biliary tract cancer (BTC) is extremely poor and only a few standard treatments are available for this condition. We performed a phase I trial to investigate the safety, immune response and anti-tumor effect of vaccination with three peptides derived from cancer-testis antigens.

**Methods:**

This study was conducted as a phase I trial. Nine patients with advanced BTC who had unresectable tumors and were refractory to standard chemotherapy were enrolled. Three HLA-A*2402 restricted epitope peptides-cell division cycle associated 1 (CDCA1), cadherin 3 (CDH3) and kinesin family member 20A (KIF20A)-were administered subcutaneously, and the adverse events and immune response were assessed. The clinical effects observed were the tumor response, progression-free survival (PFS) and overall survival (OS).

**Results:**

The three-peptide vaccination was well-tolerated up to a dose of 3 mg per peptide (9 mg total). No grade 3 or 4 adverse events were observed after vaccination. Peptide-specific T cell immune responses were observed in all patients and stable disease was observed in 5 of 9 patients. The median PFS and OS were 3.4 and 9.7 months. The Grade 2 injection site reaction and continuous vaccination after PD judgment appeared to be prognostic of OS.

**Conclusions:**

Multiple-peptide vaccination was well tolerated and induced peptide-specific T-cell responses.

**Trial registration:**

This study was registered with the University Hospital Medical Information Network Clinical Trials Registry (UMIN-CTR000003229).

## Background

Biliary tract cancer (BTC) is not a common disease worldwide, but is prevalent in East Asia and Latin America. The occurrence rate is gradually increasing and there is a high mortality rate because most cases of BTC are not diagnosed until advanced and inoperable. At this time, very few standard treatments have been established for BTC [1,2], and thus development of new treatment modalities is urgently needed. Recently, cancer vaccines using synthetic peptides have been undergoing development throughout the world, and some of them have already been shown to be safe and effective [3-12]. We have previously reported that cancer peptide vaccines are capable of inducing antigen-specific cytotoxic T cells *in vivo* and providing some clinical benefit to some patients with advanced biliary tract cancer [13]. In this study, we selected three cancer-testis antigens that were identified by using cDNA microarray technology coupled with laser microdissection and were overexpressed in nearly 100% of BTC. We then performed a phase I study to assess the safety and antigen-specific immune response of a three-peptide vaccination using the selected antigens in patients with advanced biliary tract cancer. Patients were vaccinated on a continuous basis over the long-term even if their disease had progressed. We assessed the safety of the vaccination by CTCAE v3.0 as a primary endpoint and the antigen-specific immune response and clinical effects as secondary endpoints.

## Methods

### Patient eligibility

Patients with unresectable BTC (intrahepatic bile duct cancer, extrahepatic bile duct cancer and gallbladder cancer) who were refractory to standard chemotherapy were eligible for this study. All patients were required to have an HLA-A type of A*2402. Additional inclusion criteria consisted of age between 20 and 80 years, no severe organ function impairment, white blood cell count between 2000 and 10000/mm^3^, hemoglobin > 8 mg/dL, platelet count > 100,000/mm^3^, AST and ALT < 100 IU/L, and total bilirubin < 2 mg/dl. Performance status measured by the ECOG scale was 0 to 2. It was required that there be an at least 4-week interval since the last chemotherapy. The exclusion criteria consisted of pregnancy, serious infections, severe underlying disease, severe allergic disease and a judgment of unsuitability by the principal investigator.

### Study design and endpoints

This study was conducted as a phase I trial. Patients who received standard chemotherapy under a diagnosis of inoperable BTC between June 2009 and May 2010 were invited to participate after providing their informed consent. The HLA-A genotypes of these patients were examined, and the 9 patients with an HLA-A type of A*2402 were enrolled. Three peptides were used for the vaccine, CDCA1 (VYGIRLEHF) [14], CDH3 (DYLNEWGSRF) [15] and KIF-20A (KVYLRVRPLL) [16]. These peptides were chosen from among the antigens identified by using cDNA microarray technology coupled with laser microdissection and were the most overexpressed in BTCs. The purity (>97%) of the peptides was determined by analytical high-performance liquid chromatography (HPLC) and mass spectrometry analysis. The endotoxin levels and bioburden of these peptides were tested and determined to be within acceptable levels based on the GMP grade for the vaccines (PolyPeptide or NeoMPS Inc., San Diego, CA, USA). These peptides were mixed with incomplete Freund's adjuvant (IFA; Montanide ISA51, SEPPIC),which has been used in many clinical studies, and were injected subcutaneously into the inguinal or the axicilla site. Each of the three peptides at doses of 1 mg, 2 mg or 3 mg was injected subcutaneously into three patients once a week until the 8th vaccination and every two weeks after the 9th vaccination as a monotherapy as long as possible even if the patient was judged to exhibit disease progression. The primary endpoint in this study was the assessment of toxicities caused by the vaccination based on the Common Terminology Criteria for Adverse Events version 3.0 (CTCAE v.3.0). The secondary endpoints were assessment of the immunological response, tumor response, progression-free survival (PFS) and overall survival (OS) from the 1^st^ dose given. For the image analysis, CT scan or ultrasound was performed during the pre-vaccination period and at every 4^th^ vaccination. This study was approved by the institutional review board at Tokyo Women's Medical University and was registered with the University Hospital Medical Information Network Clinical Trials Registry (UMIN-CTR number, 000003229). Informed consent was obtained from all the patients and the procedures followed were in accordance with the Declaration of Helsinki.

### Lymphocyte preparation for immunologic monitoring

The performance of the immunologic assay at the center laboratory was periodically standardized and validated by Clinical Laboratory Improvements Amendments (CLIA) and the International Conference on Harmonization of Technical Requirements for Registration of Pharmaceuticals for Human use (ICH) guidelines. PBLs were obtained from the patients at the pre-vaccination period and after every 4^th^ vaccination. Peripheral blood was taken by venipuncture, collected in an EDTA tube and transferred to the center laboratory within 24 hrs at room temperature. Within 24 hrs of blood collection, PBLs were isolated with Ficoll-Paque Plus (GE Healthcare Bio-sciences, Piscataway, NJ) density gradient solution and were stored at -80°C in cell stock media (Juji Field) without serum at 5 × 10^6^ cells/ml. After thawing, the cell viability was confirmed to be more than 90% by trypan-blue dye.

### Enzyme-linked immunospot (ELISPOT) assay

The peptide-specific CTL response was estimated by ELISPOT assay following *in vitro* sensitization. Frozen PBMCs derived from the same patient were thawed at the same time, and the viability was confirmed to be more than 90%. PBMCs (5 × 10^5^/ml) were cultured with 10 μg/ml of the respective peptide and 100 IU/mL of IL-2 (Novartis, Emeryville, CA) at 37°C for two weeks. The peptide was added to the culture at day 0 and day 7. Following CD4+ cell depletion by a Dynal CD4 Positive Isolation Kit (Invitrogen, Carlsbad, CA), an IFN-γ ELISPOT assay was performed using a Human IFN-γ ELISpot PLUS kit (MabTech, Nacka Strand, Sweden) according to the manufacturer’s instructions. Briefly, HLA-A*2402-positive B-lymphoblast TISI cells (IHWG Cell and Gene Bank, Seattle, WA) were incubated with 20 μg/ml of vaccinated peptides overnight, and then the residual peptide in the media was washed out to prepare peptide-pulsed TISI cells as the stimulation cells. Prepared CD4- cells were cultured with peptide-pulsed TISI cells (2 × 10^4^ cells/well) at a 1/1, 1/2, 1/4 or 1/8 mixture ratio of responder cells to stimulator cells (R/S ratio) on a 96-well plate (Millipore, Bedford, MA) at 37°C overnight. Non-peptide-pulsed TISI cells were used as a negative control for the stimulator cells. To confirm IFN-γ productivity, the responder cells were stimulated with PMA and ionomycin (3 μg/ml) overnight, then applied to an IFN-γ ELISPOT assay (2.5 × 10^3^ cells/well) without stimulator cells. All ELISPOT assays were performed in triplicate wells. The plates were analyzed by an automated ELISPOT reader, ImmunoSPOT S4 (Cellular Technology, Ltd., Shaker Heights, OH) and ImmunoSpot Professional Software Version 5.0 (Cellular Technology, Ltd.). The number of peptide-specific spots was calculated by subtracting the number of spots in the control well from the number of spots in each of the wells with peptide-pulsed TISI cells. The sensitivity of our ELISPOT assay was estimated as an approximately average level by an ELISPOT panel of the Cancer Immunotherapy Consortium [CIC (http://www.cancerresearch.org/cic/tools-initiatives/immune-assay-proficiency-harmonizationpanels)].

### Flow cytometry assay

Conventional two-color analysis was performed with FITC-conjugated anti-human CXCR3 mAb plus PE-conjugated anti-human CCR4 mAb (R&D Systems, Minneapolis, MN) in order to assess the host immune condition of the type 1/type 2 subsets.

### Statistical analysis

PFS and OS rates were analyzed using the Kaplan-Meier method. Statistical analyses of prognostic factors were done using the log-rank test. A p-value less than 0.05 was considered to indicate a statistically significant difference. All statistical analyses were conducted using IBM SPSS Statistics 21 (IBM, USA).

## Results

### Patient characteristics

Nine patients (4 males and 5 females; median age: 61.4 years; range: 38-76) whose HLA type was A*2402 were enrolled in this study (Table 1). Their primary tumor site was the intrahepatic bile duct in 4 cases, the extrahepatic bile duct in 3 cases, and the gallbladder in 2 cases. They had several metastases to the liver, lungs, lymph nodes, peritoneum and bone. Previous therapies consisted of operation, gemcitabine (GEM), cisplatin (CDDP), tegafur-gimeracil-oteracil potassium (TS-1), carboplatin (CBDCA) or docetaxcel (DTX). One patient dropped out after the 1^st^ follow-up study because of another disease and 4 patients elected to stop vaccination at the time of PD judgment. Four patients decided to continue the vaccination as long as possible after PD judgment.

**Table 1:** Patient characteristics

**Patients**	**Age/Sex**	Tumor site*		Prior therapy**	**Peptide**
		**Primary**	**Metastases**		**(mg)**
1	38/F	IBD	Lung, bone	Ope, GEM, CBDCA, TS-1, DTX	1
2	69/M	GB	Liver, LN	Ope, GEM, TS-1	1
3	60/F	GB	Liver, LN	Ope, GEM, TS-1	1
4	66/F	IBD	Liver, lung, LN, bone	Ope, GEM, TS-1	2
5	75/M	IBD	Lung	Ope, GEM, TS-1	2
6	61/F	IBD	Liver, LN, peritoneum	GEM, TS-1, CDDP	2
7	46/M	EBD	Liver, LN	Ope, GEM, TS-1	3
8	76/M	EBD	Lung	Ope, GEM, TS-1	3
9	62/F	EBD	Lung	Ope, GEM, TS-1	3

*IBD: intrahepatic bile duct; GB: gallbladder; EBD: extrahepatic bile duct; LN: lymph node.

**Ope: operation; GEM: gemcitabine; CBDCA: carboplatin; TS-1: tegafur-gimeracil-oteracil potassium; DTX: docetaxcel; CDDP: cisplatin.

### Assessment of toxicity

Toxicity was assessed by CTCAE v3.0 (Table 2). Four of 9 patients developed grade 1 injection site reaction and 5 developed grade 2 injection site reaction. Low hemoglobin, WBC, lymphopenia, neutrophil and platelet counts were observed before the 1^st^ vaccination and were not worsened throughout the vaccination term. No other adverse events were seen throughout the peptide vaccination. Therefore, the multiple-peptide vaccine therapy was well-tolerated up to a dose of 3 mg for each peptide, or 9 mg total.

**Table 2:** Summary of adverse events

**Adverse events**	**Total (%)**	**Grade 1 (%)**	**Grade 2 (%)**	**Grade 3 (%)**	**Grade 4 (%)**
Hemoglobin	5 (55.6)	3 (33.3)	2 (22.2)	0	0
WBC	2 (22.2)	0	2 (22.2)	0	0
Lymphopenia	3 (33.3)	2 (22.2)	0	1 (11.1)	0
Neutrophil	2 (22.2)	0	2 (22.2)	0	0
Platelet	3 (33.3)	3 (33.3)	0	0	0
Injection site reaction	9 (100)	4 (44.4)	5 (55.6)	0	0

### Antigen-specific immune response

**Figure 1:**
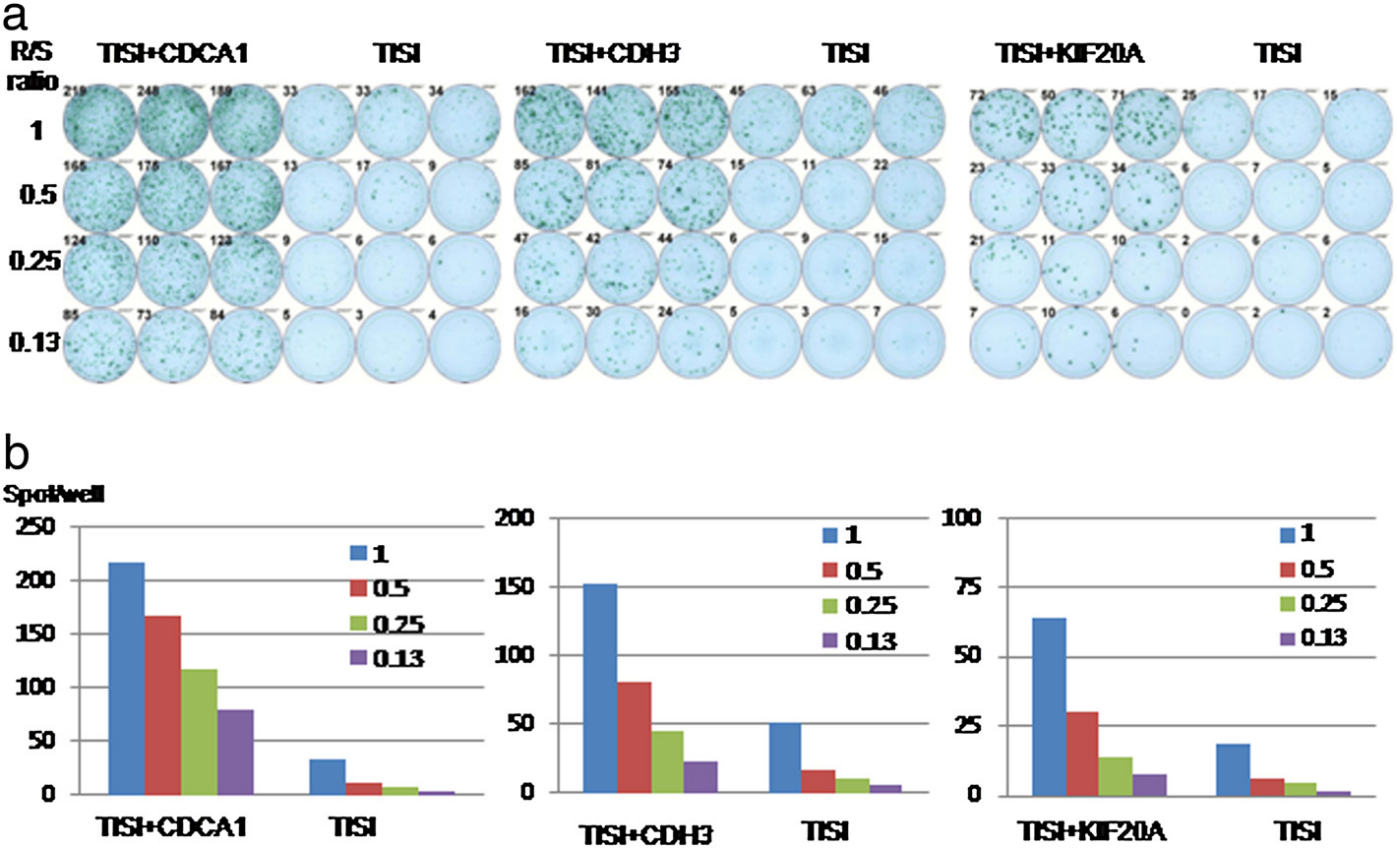
**Immunological monitoring assay of T cell response to CDCA1, CDH3 and KIF20A in patient 8. (a)** IFN-γ ELISPOT assay for CDCA1, CDH3 and KIF20A. **(b)** IFN-γ positive spots at several R/S ratio.

**Figure 2:**
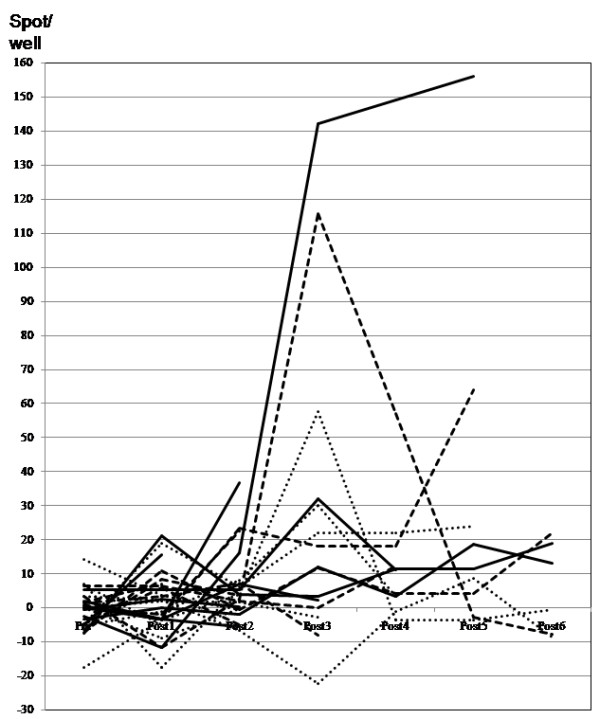
**Kinetics of the peptide-specific T cell response after vaccination.** Specific spots were counted as shown in Additional file 1: Figure S1 to CDCA1 (solid line), KIF20A (dotted line) and CDH3 (dashed line). The R/S ratio was 0.50.

Antigen-specific immune response was assessed by the ELISPOT assay. In the assay, CDCA1, CDH3 and KIF20A peptides-specific IFN-γ spots were observed in 9 of 9 patients (Figure 1). The response to every antigen in every patient was determined using our algorithm (Additional file 1: Figure S1) is summarized in Tables 3 and 4. The number of peptide-specific IFN-γ spots gradually increased with the number of vaccinations (Figure 2). These immune responses were not found for all antigens or for all patients after 8 vaccinations when the clinical assessments were done, but they were observed after the judgment of PD in some patients with continuous peptide vaccination. The group receiving 3 mg of each peptide tended to show stronger CTL induction throughout the course of this study.

**Table 3:** Summary of clinical outcome and immunological response

**Patients**	**No. of vaccine**	Clinical response*	**PFS (days)**	**OS (days)**	**After vaccine**	ISR** (Grade)	**Peptide-specific CTL**		
							**CDCA1**	**CDH3**	**KIF20A**
1	16	SD	212	310	No	2	0	1+	1+
2	8	PD	53	134	No	1	2+	0	1+
3	18	SD	127	205	No	1	1+	2+	1+
4	19	SD	505	659	Yes	2	1+	0	1+
5	27	SD	225	290	No	2	1+	1+	3+
6	10	SD	101	101		1	1+	1+	0
7	13	PD	64	164	Yes	1	2+	1+	2+
8	24	PD	64	353	Yes	2	3+	3+	3+
9	25	PD	57	380	Yes	2	2+	3+	2+

*SD: stable disease; PD: progressive disease.

**ISR: injection site reaction.

**Table 4:** CTL response to CDCA1, CDH3 and KIF20A

**Peptide dose**	**No.**	**Course**	**CTL response**			
			**CDCA1**	**CDH3**	**KIF20A**	**Positive control**
1 mg	1	Pre	-	+	-	+++
		Post 1	-	-	+	+++
		Post 2	-	-	-	+++
	2	Pre	-	-	-	+++
		Post 1	++	-	+	+++
	3	Pre	+	-	-	+++
		Post 1	NA*	NA	NA	+++
		Post 2	-	+	-	+++
		Post 3	+	++	-	+++
		Post 4	+	-	+	+++
2 mg	4	Pre	+	-	+	+++
		Post 1	-	-	+	+++
		Post 2	+	-	+	+++
		Post 3	-	-	-	+++
	5	Pre	-	-	-	+++
		Post 1	-	-	-	+++
		Post 2	-	-	+	+++
		Post 3	+	-	+++	+++
		Post 4	-	-	++	+++
		Post 5	+	+	-	+++
		Post 6	-	-	-	+++
	6	Pre	-	+	-	+++
		Post 1	+	+	-	+++
		Post 2	+	+	-	+++
3 mg	7	Pre	NA	NA	NA	+++
		Post 1	-	-	-	+++
		Post 2	++	+	++	+++
	8	Pre	-	-	+	+++
		Post 1	-	+	-	+++
		Post 2	++	+	++	+++
		Post 3	+++	+++	+	+++
		Post 5	+++	+++	+++	+++
	9	Pre	+	-	+	+++
		Post 1	++	+	+	+++
		Post 2	-	+	-	+++
		Post 3	-	+++	+	+++
		Post 4	++	-	-	+++
		Post 6	++	+	++	+++

*NA: not analyzed.

### Clinical response

**Figure 3:**
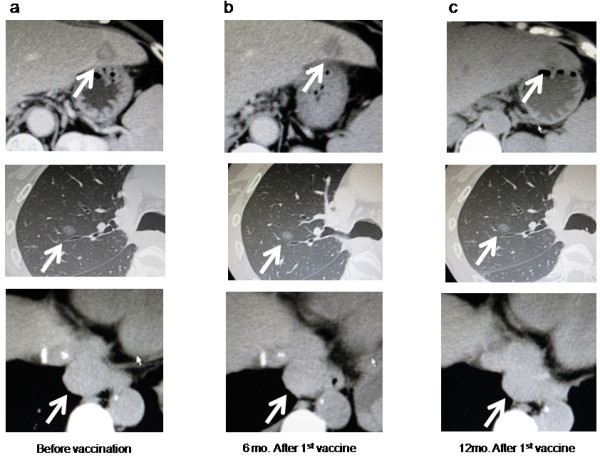
**Clinical response assessment in the longest-surviving patient, patient 4.** CT imaging of the liver, lung and para-aortic lymph node metastasis before vaccination **(a)**, 6 months after 1st vaccination **(b)** and 12 months after 1st vaccination **(c)**. The tumor sizes were stable across the 505 days after 1st vaccination.

**Figure 4:**
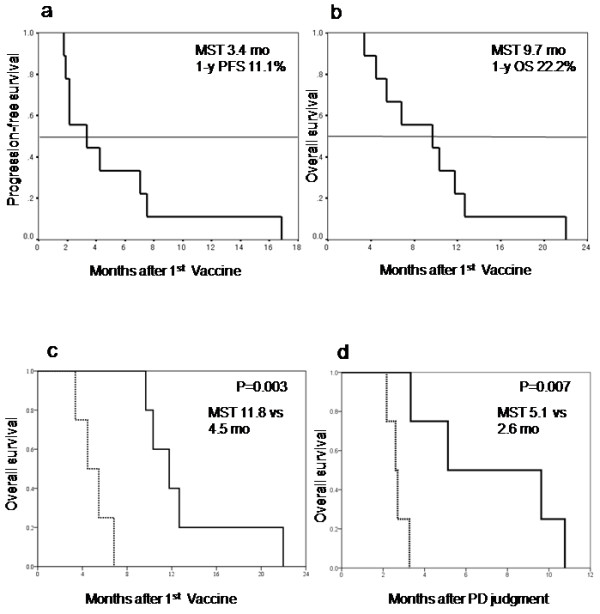
**Progression-free survival and overall survival in all enrolled patients. (a)** Progression-free survival after 1st vaccination. The MST was 3.4 months and the 1-year PFS ratio was 11.1%. **(b)** Overall survival after the 1st vaccination. The MST was 9.7 months and the 1-year OS ratio was 22.2%. **(c)** Overall survival of the patients with Grade 2 ISR (solid line) compared to that of the patients with Grade 1 ISR (dotted line). There was a statistically significant difference between these 2 groups (p = 0.003). **(d)** Overall survival of the patients receiving continuous vaccination (solid line) compared to that of the patients who stopped the vaccination at the determination of PD (dotted line). There was a statistically significant difference between these 2 groups (p = 0.007).

As shown in Tables 3, 5 patients had stable disease (SD) and 4 had progressive disease (PD) as judged after the 8^th^ vaccination. The 8 patients continued to be administered the vaccination and 4 of them continued to receive the vaccination for as long as possible, even if their disease developed PD. The vaccinations eventually achieved disease stability in the patients who received the long-term vaccination (Figure 3 shows the CT scan of patient 4), but in the end their diseases progressed, and they had all died within 2 years of the 1^st^ vaccination. Their median progression-free survival (PFS) for all patients after the first vaccination was 3.4 months (95% CI: 0-7.0) and the 1 year PFS was 11.1% (Figure 4a). The median overall survival (OS) for all patients was 9.7 months (95% CI: 1.4-18.0) and the 1 year OS was 22.2% (Figure 4b).

**Table 5:** Prognostic factors of PFS or OS

**Factors**	**PFS**	**OS**
Sex (male/female)	0.426	0.302
Age (≧61/<61)	0.706	0.084
CRP (≧1.5/<1.5)	0.654	0.832
Hemoglobin (≧12/<12)	0.351	0.435
Lymphocyte (%) (≧27/<27)	0.145	0.132
Lymphocyte (number) (≧1500/<1500)	0.488	0.900
CDCA1 CTL spots (≧2+/<2+)	0.004	0.870
CHD3 CTL spots (≧2+/<2+)	0.235	0.611
KIF20A CTL spots (≧2+/<2+)	0.486	0.840
CXCR3 + CCR4- T cells (≧8%/<8%)	0.046	0.966
CXCR3 + CCR4- T cells (up/down)	0.007	0.604
Injection site reaction (≧Grade2/<Grade2)	0.145	0.003
Continuous vaccination after PD (Yes/No)	-	0.007

### Univariate analysis of the prognostic factors

The results of the univariate analysis of the prognostic factors are described in Table 5. The patients who developed grade 2 local skin reaction at the vaccination site showed a median OS of 11.8 months (95% CI: 8.7-14.9). This was better than the OS of patients with grade 1 local skin reaction of 4.5 months (95% CI: 2.4-6.5). There was a significant difference between these 2 groups (p = 0.003) (Figure 4c). The OS of the patients with continuous vaccinations was also better than that of the patients who stopped the vaccination as their disease progressed. The OS of the patients with continuous vaccinations was 5.1 months (95% CI: 0-11.3) and that of the patients who stopped the vaccination was 2.6 months (95% CI: 2.1-3.1). There was a statistically significant difference between these 2 groups (p = 0.007) (Figure 4d).

### Analysis of the relation between better PFS and the type 1 host immune condition

**Figure 5:**
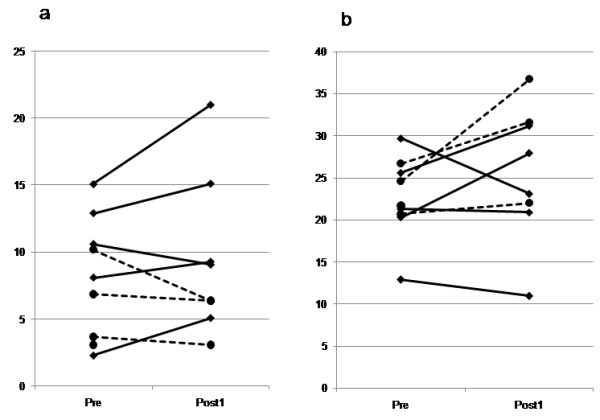
**Kinetics of type 1 T cell population and type 2 T cell population after vaccination. (a)** The population of CXCR3 + CCR4- type 1 T cells increased in most of the patients with stable disease (SD) (solid line) and decreased all the patients with progressive disease (PD) (dashed line) after 4 vaccinations. **(b)** The population of CXCR3-CCR4+ type 2 T cells was decreased in most of the patients with SD (solid line) and increased in all the patients with PD (dashed line) after 4 vaccinations.

The type 1 host immune condition was analyzed based on the ratio of the CXCR3 + CCR4- T cell population (mean: 8.1%). The patients with a CXCR3 + CCR4- T cell population of more than 8% showed longer PFS and the patients whose CXCR3 + CCR4- T cell population increased after the 4^th^ vaccinations also showed longer PFS (Table 5). Most of the patients with stable disease (SD) showed an increase in the CXCR3 + CCR4- type 1 T cell population (Figure 5a) and a decrease in the CXCR3-CCR4+ type 2 T cell population after the 4^th^ vaccination (Figure 5b). In contrast, all the patients with progressive disease (PD) showed a decrease in the CXCR3 + CCR4- T cell population and an increase in the CXCR3-CCR4+ T cell population after the 4^th^ vaccination. Therefore, the type 1 host immune condition was suspected to be an important factor for achieving disease stability through the induction of peptide-specific CTLs *in vivo*.

## Discussion

It is difficult to detect BTCs in the early stage, and this difficulty is partly responsible for the poor prognosis of the disease. Operation is the most effective treatment, but recurrence or metastasis occurs at a high rate following curative operation. Other than operation, chemotherapy is the only therapy currently available. Nevertheless, GEM plus CDDP, which is the best choice of chemotherapy for BTC, only yields a median overall survival of 11.7 months and progression-free survival of 8.0 months. However, a median overall survival of 9.7 months was realized using a three-peptide vaccination after the failure of standard chemotherapies, and thus this modality might have potential for improving the OS in patients with BTC.

In this study, we selected three new peptides for BTC. CDCA1 is a molecular linker between the kinetochore attachment site and the tubulin subunits. CDH3 is a cell-cell adhesion molecule and takes part in the signal transduction for cell growth and differentiation. KIF20A is a conserved motor domain that binds to microtubules. We previously reported on other combinations of peptides for vaccination in patients with BTC. We found that DEPDC1 and LY6K strongly induced antigen-specific CTLs after the 4th vaccination. In contrast, IMP3 and TTK induced CTLs only weakly and late after the vaccination. The three-peptide vaccination in this study also showed a delayed induction of peptide-specific CTLs. We speculate that this might have been due to differences in the abilities of the peptides to induce a host immune response. In these cases, most of the patients were judged as having PD before the strong CTLs were induced *in vivo*. The protocol of this study permitted the continuation of vaccination after the diagnosis of PD, and the patients who continued vaccination after the start of PD had a strong CTL induction and showed better prognosis compared with other patients who stopped the vaccination at the time of PD judgment. Therapeutic cancer vaccination seems to show a delayed clinical effect [17] and the early discontinuance of vaccination might cause a misappraisal of the true capacity of the cancer vaccine. Therefore, there may be need of a further clinical study in which the vaccination is continued for the long term even after a diagnosis of PD in the early stage of study, because the induction of CTLs was often fairly slow.

Several phase III randomized studies of cancer vaccines have been performed [18], but very few of them were successful [19]. The clinical efficacy of cancer vaccines is currently limited because of the immune checkpoint. Anti-CTLA-4 mAb (ipilimimab) [20], anti-PD-1 [21,22] and PD-L1 [23] have shown promising results in some clinical studies. Although the blockage of the immune checkpoint itself is an effective therapy, it also seems to be necessary to administer the cancer antigen-specific CTLs. Cancer peptide vaccines could induce antigen-specific CTLs *in vivo*, so the combination of a cancer vaccine and immune checkpoint blockade would be a more successful anti-cancer strategy in the future. Another approach might be to improve the immune condition of the host. A proper number of lymphocytes, especially type 1 T cells, seems to be needed to acquire a good immune response, which in turn has been associated with a better prognosis [24,25]. In order to ensure the success of clinical trials, a new classification method or biomarker is needed to stratify patients according to their immune condition [26-30].

In this report, a new three-peptide vaccine was shown to be safe and to elicit an effective immune response in patients with advanced biliary tract cancer. No patients exhibited a CR or PR, but it was suggested that the OS could be extended by continuous administration of this vaccination. In order to establish this immunotherapy as the standard therapy for biliary tract cancer, it will be necessary to assess the survival improvement in a phase II/III randomized trial with an appropriate number of subjects. We have reported 4 peptides previously and 3 new peptides in this study. All 7 of these peptides could be used simultaneously for patients with advanced BTC, or one or more of them could be selected for patients in an adjuvant setting after operation and examination of the antigen expression profile in their tumor cells.

## Conclusions

We have shown that a cancer peptide vaccine therapy using a mixture of three peptides was well tolerated and could induce peptide-specific CTLs in patients with advanced BTC. The peptide vaccine was found to have a sufficient effect on survival to merit further evaluation in clinical trials.

## Abbreviations

BTC: Biliary tract cancer; CBDCA: Carboplatin; CDCA1: Cell division cycle associated 1; CDDP: Cisplatin; CDH3: Cadherin 3; CTCAE: Common terminology criteria for adverse events; CTL: Cytotoxic T lymphocyte; DTX: docetaxcel; ELISPOT: Enzyme-linked immunospot; GEM: Gemcitabine; HPLC: High-performance liquid chromatography; IFA: Incomplete Freund's adjuvant; KIF20A: Kinesin family member 20A; OS: Overall survival; PBL: Peripheral blood lymphocyte; PFS: Progression-free survival; SD: Stable disease; TS-1: Tegafur-gimeracil-oteracil potassium.

## Competing interests

The authors declare that they have no competing interests.

## Authors’ contributions

AA participated as the principle investigator of the study and drafted the manuscript. NT and NM participated the acquisition of data. KT coordinated the analysis of immunological data. YK and RO participated in the design and coordination of the study. TO and MY participated in the study supervision. All authors read and approved the final manuscript.

## Supplementary Material

Additional file 1 Algorithm of the assessment of CTL response to antigen. Click here for file
