# Supplementary figures and images for: Phase I clinical trial of multiple-peptide vaccination for patients with advanced biliary tract cancer
Source: J Transl Med. 2014 Mar 7;12:61. doi: 10.1186/1479-5876-12-61 (PMC4015445; doi:10.1186/1479-5876-12-61)

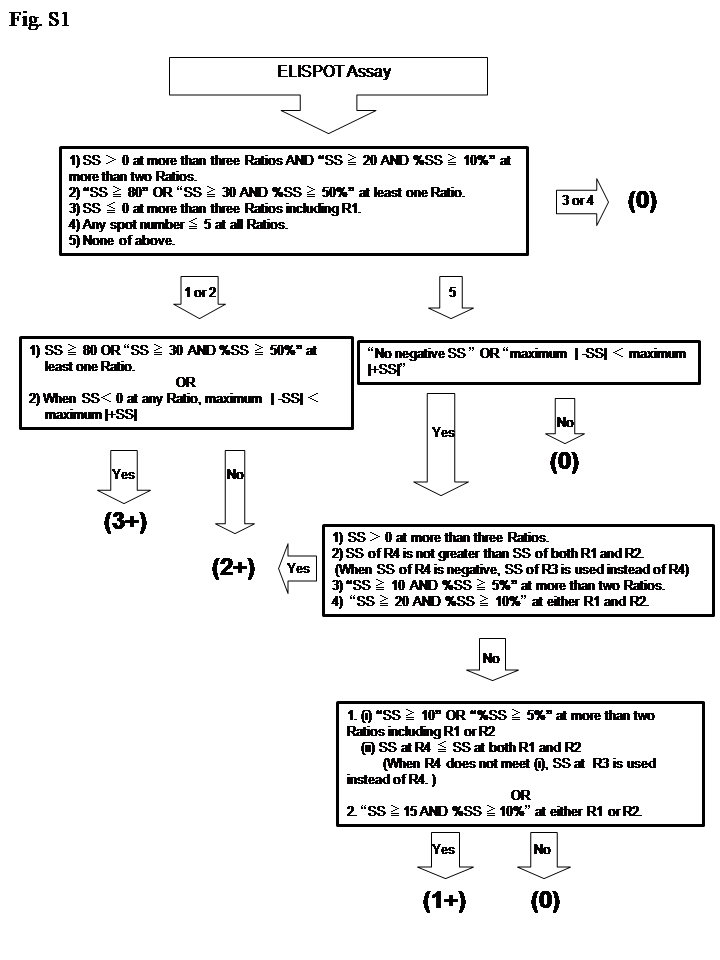

Supplement: Additional file 1 — Algorithm of the assessment of CTL response to antigen. [file 1479-5876-12-61-S1.tiff]
